# ABO blood group is involved in the quality of the specific immune response anti-SARS-CoV-2

**DOI:** 10.1080/21505594.2021.2019959

**Published:** 2021-12-30

**Authors:** Sergio Gil-Manso, Iria Miguens Blanco, Bruce Motyka, Anne Halpin, Rocío López-Esteban, Verónica Astrid Pérez-Fernández, Diego Carbonell, Luis Andrés López-Fernández, Lori West, Rafael Correa-Rocha, Marjorie Pion

**Affiliations:** a Laboratory of Immune- Gregorio Marañón Health Research Institute (IiSGM), Gregorio Marañón University General Hospital, Madrid, Spain; b Department of Emergency, Gregorio Marañón University General Hospital, Madrid, Spain; c Department of Pediatrics, Alberta Transplant Institute and Canadian Donation and Transplantation Research Program; University of Alberta, Edmonton, Alberta, Canada; d Laboratory Medicine & Pathology, University of Alberta, Edmonton, Alberta, Canada; e Department of Hematology, Gregorio Marañón University General Hospital, Madrid, Spain; f Service of Pharmacy, Gregorio Marañón Health Research Institute (IiSGM), Gregorio Marañón University General Hospital, Spanish Clinical Research Network (SCReN), Madrid, Spain; g Medical Microbiology & Immunology, Surgery, and Laboratory Medicine & Pathology; University of Alberta, Edmonton, Alberta, Canada

**Keywords:** ABO group, memory T-cell response, humoral immune response, individual factors, SARS-CoV-2

## Abstract

Since December 2019, the coronavirus disease 2019 (COVID-19), caused by the severe acute respiratory syndrome coronavirus 2 (SARS-CoV-2), has spread throughout the world. To eradicate it, it is crucial to acquire a strong and long-lasting anti-SARS-CoV-2 immunity, by either natural infection or vaccination. We collected blood samples 12–305 days after positive polymerase chain reactions (PCRs) from 35 recovered individuals infected by SARS-CoV-2. Peripheral blood mononuclear cells were stimulated with SARS-CoV-2-derived peptide pools, such as the spike (S), nucleocapsid (N) and membrane (M) proteins, and we quantified anti-S immunoglobulins in plasma. After 10 months post-infection, we observed a sustained SARS-CoV-2-specific CD4+ T-cell response directed against M-protein, but responses against S- or N-proteins were lost over time. Besides, we demonstrated that O-group individuals presented significantly lower frequencies of specific CD4+ T-cell responses against Pep-M than non O-group individuals. The non O-group subjects also needed longer to clear the virus, and they lost cellular immune responses over time, compared to the O-group individuals, who showed a persistent specific immune response against SARS-CoV-2. Therefore, the S-specific immune response was lost over time, and individual factors might determine the sustainability of the body’s defenses, which must be considered in the future design of vaccines to achieve continuous anti-SARS-CoV-2 immunity.

## Introduction

Since December 2019, a new severe acute respiratory syndrome coronavirus 2 (SARS-CoV-2), causing coronavirus disease 2019 (COVID-19), has spread worldwide, triggering various clinical manifestations in infected patients, such as anosmia, dry cough, fatigue, fever, diarrhea, and pneumonia [1]. The SARS-CoV-2 pandemic poses a serious health threat to the global population. The most effective way to protect the population without suffering quarantine, would be to achieve widespread anti-SARS-CoV-2 immunity, after either natural infection or vaccination. Information about the immune system’s sustainability or efficiency in fighting the virus is central to improving patient management [2,3]. Indeed, even people with mild symptoms may experience long-term sequelae and, possibly, immune dysregulation, and it is unknown if these long-term symptoms could be associated with re-infection or future pathogenesis [4–6].

Markers of the protective humoral response, such as total anti-SARS-CoV-2 immunoglobulins and neutralizing antibodies, have been observed to decrease in convalescent individuals, even though a potential long-lasting humoral B-cell memory subset was detected [7–9]. The loss of humoral immunity or the acquisition of mutations in the viral genome have been associated with increased cases of COVID-19 recurrence [10–12]. These recurrences can be due to re-infection or viral re-activation; in both cases, immunity is at the center of viral clearance.

Less is known about long-term cellular protection, which is pivotal for resolving viral infections and developing long-lasting immunity. Positive and promising results have suggested that cellular immunity can be generated during SARS-CoV-2 infection [13–15], as demonstrated in SARS coronavirus infection, where memory T-cells could be detected 11 years after infection [16]. The detection of these specific T-cells comprises evidence for potential preexisting immunity mediated by T-cells cross-reactive to human common-cold coronaviruses, which might protect against SARS-CoV-2 infection [17–19]. Induced T-cell immunity also appears to play a critical role in SARS-CoV-2 clearance, with studies reporting strong T-cell responses in acute infection up to the convalescence phase [18,20]. Therefore, we studied the persistence of the antigen-specific response in individuals that had recovered from COVID-19, along with possible individual factors related to the duration and intensity of the immune response. Such information could help in the stratification of individuals according to re-infection risk factors, in order to prioritize those at high risk for immunization.

## Patients and materials/methods

### Patients and blood samples

Blood samples and questionnaire data regarding donor characteristics during COVID-19 infection from SARS-CoV-2 convalescent donors were collected at the General University Hospital Gregorio Marañón, Spain, from 6/2020 to 12/2020. Informed consent was obtained under the Declaration of Helsinki protocol. The study was approved by the local ethics committee and performed according to their guidelines (COV1-20-007). SARS-CoV-2 infection was confirmed by a PCR test after a nasopharyngeal swab. SARS-CoV-2 donors were recruited among health workers of the General University Hospital Gregorio Marañón in Madrid who had been infected by SARS-CoV-2 between March and December 2020. Samples were collected at a single time point, between 12 days post-positive PCR (P-PCR+) and 305 days P-PCR+ (Table 1). The Spanish health protocol for infected individuals’ follow-up, used in our hospital at the time of samples collection, was to test health workers weekly after the first PCR+. Therefore, the days between PCR+ and negative PCR were the number of days between the first positive PCR after symptoms appear and the first negative PCR during the follow-up. Nevertheless, during two months (November and December 2020), the protocol was changed, and the patients were forced to quarantine for 14 days after testing positive, without repeating the PCR after quarantine. Thus, the negative PCR result from these individuals could not be obtained, and therefore, of the 35 patients included in the study, only 27 had the date for the negative PCR. Whole blood was labeled for surface markers to determine the absolute numbers of lymphocytes or CD4+ and CD8 + T cells (Table S1: Whole blood labeling section). After surface labelling, red blood cells were lysed using RBC Lysis/Fixation Solution (BioLegend, San Diego, CA, U.S.). Absolute numbers of cellular subsets were determined using Flow-Count Fluorospheres (Beckman Coulter, Nyon, Switzerland). Flow cytometry analyzed surface markers using a MACSQuant Analyzer 16 cytometer (Miltenyi Biotec, Bergisch Gladbach, Germany). PBMCs were then isolated by density gradient centrifugation. The serum was separated by centrifugation, and the supernatant was stored at −80°C. The classification of symptoms was based on responses to a questionnaire by individual donors. The score (Asymptomatic/Mild/Moderate) was based on the criteria of the WHO Working Group on the Clinical Characterization and Management of COVID-19 infection [21]. The characteristics of the SARS-CoV-2-recovered donors are detailed in Table 1.

**Table 1.** Demographic and clinical characteristics of the COVID-19 convalescent patients

	**Patients (n=35)**
**Age (years), median (range)**	40 (25–62)
**Gender, n (%)**	
Male	11 (31.4)
Female	24 (68.6)
**Blood Type, n (%)**	
A (Rh+/Rh-)	15 (42.9)/1 (2.9)
B (Rh+/Rh-)	2 (5.7)/1 (2.9)
AB (Rh+/Rh-)	2 (5.7)/0 (0.0)
O (Rh+/Rh-)	10 (28.6)/4 (11.4)
**Comorbidities, n (%)**	
Current smoker/ex-smoker	3 (8.6)/4 (11.4)
Asthma	3 (8.6)
Heart disease	2 (5.7)
Obesity	1 (2.9)
Hypertension	1 (2.9)
Epilepsy	1 (2.9)
Psoriasis	1 (2.9)
Sleep apnea	1 (2.9)
Fibromyalgia	1 (2.9)
Diabetes	0 (0.0)
Liver disease	0 (0.0)
Kidney disease	0 (0.0)
WHO clinical progression scale, n (%)*	
Mild, asymptomatic (1)	4 (11.4)
Mild, symptomatic independent (2)	29 (82.9)
Moderate, no oxygen therapy (4)	1 (2.9)
Moderate, oxygen by mask or nasal prongs (5)	1 (2.9)
**Symptoms during COVID-19, n (%)**	
Fatigue	19 (54.3)
Myalgia	19 (54.3)
Anosmia	16 (45.7)
Fever (≥38)	14 (40.0)
Headache	14 (40.0)
Ageusia	13 (37.1)
Cough	13 (37.1)
Diarrhea	10 (28.6)
Dyspnea	9 (25.7)
Arthralgia	5 (14.3)
Nausea or vomiting	5 (14.3)
Fever (<38)	3 (8.6)
Pneumonia	3 (8.6)
Dizziness	3 (8.6)
Tachycardia	3 (8.6)
Sore throat	2 (5.7)
Conjunctivitis	1 (2.9)
Congestion	1 (2.9)
Skin rash	1 (2.9)
**Treatment, n (%)**	
Antibiotics	8 (22.9)
Hydroxychloroquine	6 (17.1)
Nonsteroidal anti-inflammatory drug	6 (17.1)
Anticoagulants	4 (11.4)
Steroids	2 (5.7)
Antiretroviral therapy	1 (2.9)
**Days from PCR+ to PCRneg, median (range)†**	17 (6–30)
**Days from PCR+ to data analysis, median (range)**	154 (12–305)

### Stimulation with SARS-CoV-2 peptide pools

The peptide stimulation protocol was performed on isolated PBMCs. SARS-CoV-2 PepTivator peptide pools (Miltenyi Biotec), mainly consisting of 15-mer sequences with 11 amino acids (aa), were used. The peptide pool for the Spike-protein (Pep-S) contained the sequence domains aa 304–338, 421–475, 492–519, 683–707, 741–770, 785–802, and 885–1273. This peptides’ pool covers 3 regions of the receptor-binding domain of the Spike-protein but does not cover the entire Spike-protein. The peptide pools for the membrane glycoprotein (Pep-M) or the nucleocapsid phosphoprotein (Pep-N) mainly consisted of 15-mer sequences with 11 aa overlap, covering the complete sequence of the M or N protein. Two positive controls for activation were used: PepTivator against cytomegalovirus (Pep-CMV, Miltenyi Biotec), which consisted of 15-mer peptides with 11 amino acids, covering the complete sequence of the pp65 protein of human cytomegalovirus, and CytoStim (Miltenyi Biotec), an antibody-based component that acts similarly to a superantigen of the T-cell receptor. Negative controls were left non-treated (NT). PBMCs were prepared from EDTA collection tubes (Vacutainer® K2E, BD) by density gradient centrifugation. A total of 1.5 × 10^6^ PBMCs were stimulated with 1 µg/mL of peptide pools for 6 h in TexMACSTM GMP Medium (Miltenyi Biotec), supplemented with 5% AB human serum (Sigma-Aldrich, St. Gallen, Switzerland). Brefeldin A (10 µg/mL, Sigma-Aldrich) was added at the beginning of the stimulation.

### Staining for intracellular cytokines and cell surface markers after PBMC stimulation with SARS-CoV-2 peptide pools

**Figure 1.**
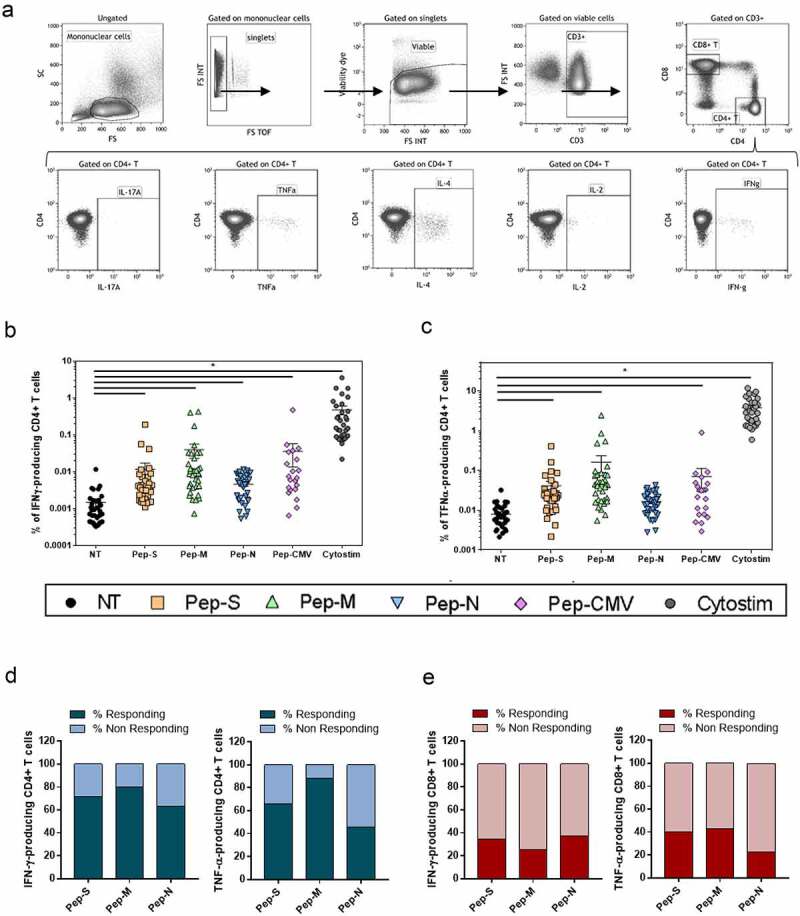
Gating strategy and specific memory T-cell responses to SARS-CoV-2-derived peptide pools in 35 convalescent patients.

Peptide-specific T-cells were characterized after 6 h of stimulation by cell surface and intracellular cytokine staining. Briefly, cells were surface-stained, stained with viability dye, fixed/permeabilised, and intracellularly stained (antibodies listed in Table S1; Specific cellular T response assay section). The cells were then analyzed by flow cytometry, using a Gallios cytometer (Beckman Coulter). All the cytometry data were analyzed using the Kaluza software (Beckman Coulter). The gating strategy applied for the analyses of flow cytometry-acquired data is provided in Figure 1 and Figure S1.

(a) Gating strategies to define SARS-CoV-2-specific CD4+ T-cells. Representative examples of flow cytometry plots of SARS-CoV-2-specific CD4+ T-cells after 6 h stimulation with Spike (Pep-S). PBMCs were isolated and stimulated by SARS-CoV-2-derived peptide pools (Pep-S, Pep-M, and Pep-N), with CMV-derived peptides (Pep-CMV), or with CytoStim. CD3, CD4 and CD8 expression was detected by surface labeling and IL-17A, TNF-α, IL-4, IL-2 and IFN-γ expression was detected intracellularly by flow cytometry. (b) Frequencies of IFN-γ-producing CD4+ T-cells. N=35. (c) Frequencies of TNF-α-producing CD4+ T-cells. N=35. Each symbol corresponds to an individual (N=35). One-way ANOVA with multiple-comparison Kruskal–Wallis tests were used. *p < 0.05. (d) Stacked bars comparing the frequency of individuals with a specific T-cell response when cells were stimulated by Pep-S, Pep-M, or Pep-N in CD4+ T cells. Individuals with SIs greater than 2 were considered responding individuals, and those with SIs lower than 2, as non-responding individuals. The response was observed as the intracellular IFN-γ or TNF-α production in CD4+ cells. N= 35. (e) Stacked bars comparing the frequencies of individuals responding and not responding to the SARS-CoV-2-derived peptides in CD8+ T-cells. N=35.

### CMV IgG detection

The 96-well CMV IgG ELISA (Abcam, Cambridge, MA, US) was performed according to the manufacturer’s instructions. This ELISA detects human anti-cytomegalovirus IgG. The final interpretation of positivity was determined by a ratio above a threshold value given by the manufacturer: positive (ratio > 11), negative (ratio < 9), or non-defined (ratio 9–11). Quality control was performed, following the manufacturer’s instructions, on the day of testing.

### Detection of blood group antigens

To detect the presence or absence of A, B, and/or RhD antigens on red blood cells, DiaClon Anti-A, DiaClon Anti-B, DiaClon Anti-AB, and DiaClon Anti-D (Bio-Rad, Basel, Switzerland) were used, with whole blood diluted in isotonic saline solution (Braun, Hessen, Germany), according to the manufacturer’s instructions. The mix was centrifuged and then resuspended, in order to observe macroscopic agglutination.

### Testing for ABO and SARS-CoV-2 antibodies using luminex single-antigen beads

Description of the ABH antigens’ structures was already detailed [22]. ABO-A and ABO-B subtype glycans I–VI were conjugated to bovine serum albumin (BSA), as previously described [23], and an optimized protein-coupling procedure was used to link each subtype antigen to individual Luminex beads [24]. BSA-only-coupled beads were used to determine the background reactivity, while Galα1-3Galβ1-(3)4GlcNAc-R (α-Gal) BSA-coupled beads were used as a positive control [25]. Coupling was confirmed using different monoclonal antibodies, including those specific to A subtypes I–VI (clone A98, Novaclone, Immucor, Dartmouth, NS, Canada), B subtypes I–VI (clones B84 and B97, Novaclone), A/B subtype II (JTL-4) [26], and A/B subtypes III/IV (JTL-2) [26]. Bound IgM monoclonal antibody was detected with PE-labeled goat anti-mouse IgM secondary antibody (Southern Biotech, Birmingham, AL, US) [25].

SARS-CoV-2 S1 (Abcam) and RBD (Sino Biological, Wayne, PA, US) proteins were conjugated to Luminex beads using standard coupling procedures [24]. Coupling was confirmed using a rabbit IgG anti-SARS-CoV-2 Spike monoclonal antibody (Sino Biological) and PE-conjugated goat anti-rabbit IgG secondary antibody (Southern Biotech).

To detect serum ABO and SARS-CoV-2 antibodies, sera (25-fold dilution) were incubated with Luminex beads for 30 min at room temperature, washed, and then incubated with a 50-fold dilution of PE-conjugated goat anti-human IgM or IgG (both from Thermo Fisher, Waltham, MA, US) for 30 min at room temperature. Samples were acquired using a FLEXMAP 3D® Luminex system (Toronto, Canada).

### Statistical analysis

Data are displayed as means with standard error. The statistical tests used to evaluate the experiments are described within the respective figure legends. Continuous data were tested for normality of distribution using Kolmogorov–Smirnov/Shapiro–Wilk tests. Comparisons were based on the unpaired Student’s t-test or Mann-Whitney U test for parametric and nonparametric continuous data, respectively. Spearman’s rho (r) was calculated to assess the correlation between continuous data. Hochberg’s corrections were performed for multiple testing. Graphs were plotted using the GraphPad Prism 7.00 software. Statistical analyses were conducted using GraphPad Prism 7.00 and the SPSS (IBM, version 25, Armonk, NY, US) software.

## Results

### Study participants

A total of 35 individuals were recruited following recovery from COVID-19, including 29 patients with mild symptoms, two patients with moderate symptomatology, and four asymptomatic cases, according to the WHO Working Group on the Clinical Characterization and Management of COVID-19 infection [21]. The donors had documented dates for PCR positivity (PCR+) and/or PCR negativity (PCRneg). At the time of the study, they no longer presented symptoms related to COVID-19 (Table 1 shows the participants’ characteristics). No significant differences in gender or age were noted between the asymptomatic, mild, and moderate individuals. A total of 94.3% of the subjects were never hospitalized for COVID-19, while 5.7% were hospitalized with moderate symptoms (n = 2), none of whom required intensive care unit care (Table 1). The subjects’ ages ranged from 25 to 62 years (Table 1).

### SARS-CoV-2 specific memory T-cells in COVID-19 patients

To study the generation of SARS-CoV-2-specific memory T-cells against structural nucleocapside (N), spike (S) and membrane (M) proteins, the intracellular cytokine expression of the donor’s PBMCs was analyzed after 6 h of stimulation with peptide pools (Figure 1a shows the gating strategy for the CD4+ subset).

The studied cytokines were intracellular IL-2, IL-4, IL-17A, IFN-γ and TNF-α, when PBMCs were non-treated (NT) or stimulated with Pep-S, Pep-M,Pep-N, Pep-CMV or CytoStim as a positive control for cellular activation (Figure S1). The CMV stimulation results represented in all the figures were derived only from CMV-seropositive individuals (n = 21) screened using the IgG anti-CMV ELISA kit. The frequencies of IFN-γ- and TNF-α-producing CD4+ T-cells were significantly higher in stimulated conditions than in NT for all the peptides, demonstrating the presence of SARS-CoV-2-specific cells in almost all the individuals (Figure 1b and 1c, respectively). However, Pep-N did not induce a significant increase in the frequency of TNF-α-producing CD4 + T-cells (Figure 1c). As the frequency of cytokine-expressing cells was close to the NT condition in some individuals, we calculated the stimulation index (SI) for each individual, by dividing the frequency of specific T-cell response against peptides pools by the respective response in the NT control. An SI above 2 was considered to indicate a detectable response, while that below 2 corresponded to a lack of response from the individual. We observed that most individuals presented a clear and robust signal after stimulation with Pep-S, Pep-M, and Pep-CMV, but that for Pep-N was less intense (Figure S2a). On the other hand, neither SARS-CoV-2-derived nor CMV-derived peptides induced IL-17A and IL-4 responses in CD4+ or CD8+ T-cells, even though some individuals presented an IL-2-producing CD4+ T-cell SI greater than 2 when stimulated with Pep-M (Figure S2b). In terms of an SI above 2 for individuals responding to the peptides, 71.4% and 65.7% of individuals responded to Pep-S, and 80% and 88.6% responded to Pep-M, while only 62.9% and 45.7% showed a detectable response to Pep-N, according to CD4+ T-cells (considering IFN-γ and TNF-α, Figure 1d, respectively). Strikingly, fewer than 50% of individuals presented responses to any peptide pools derived from SARS-CoV-2 in the CD8+ T-cells (Figure 1eand S2c). We assume that this result was not derived from experimental bias for CD8+ T-cells, as good responses were observed for the CMV-derived peptide pool and CytoStim (Figure S2c).

In summary, Pep-M induced the strongest CD4+ T-cell memory responses, followed by Pep-S and, finally, Pep-N. Moreover, SARS-CoV-2-derived peptides induced responses in almost all the individuals tested, but mostly of the CD4+ memory T-cell type.

### Detection of SARS-CoV-2-specific memory T-cells, according to the time of viral clearance and time post-infection

Our study included individuals with histories of COVID-19, who were recruited 12 to 305 days after testing positive for SARS-CoV-2 by PCR+ (P-PCR+; up to 10 months). Two of the individuals analyzed did not respond to any of the SARS-CoV-2-derived peptides. These two individuals were asymptomatic at the moment of PCR detection, with low viral load showed by high cycle thresholds (CTs) in real-time PCR.

**Figure 2.**
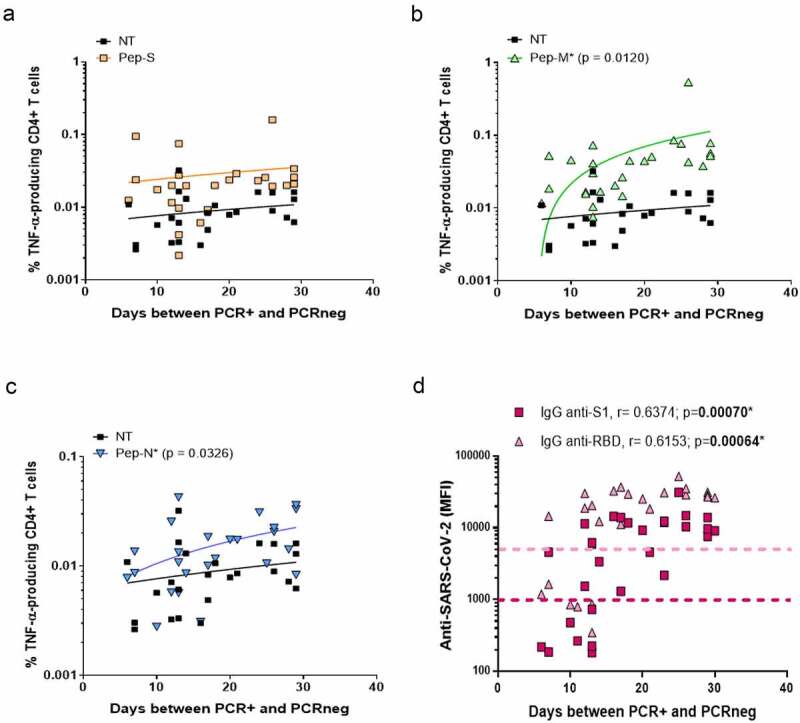
Frequencies of TNF-α-producing cells and time necessary for viral clearance.

First, we correlated the time between the beginning of the infection (the first PCR+ after the appearance of symptoms) and the end of the infection (the first PCRneg after COVID-19), corresponding to the period needed for viral clearance, with the frequency of cells responding to SARS-CoV-2-derived peptide pools. Patients recovered from the viral infections 6–30 days after the detection of the virus. No correlation was observed for the Pep-S stimulation (Figure 2a). However, the longer the time of active infection, the higher the TNF-α-specific response to Pep-M and Pep-N (p = 0.0120 and p = 0.0326, Figure 2band Figure 2c, respectively). It has already been noted that anti-SARS-CoV-2 immunoglobulins decrease in convalescent individuals after several months [27]; however, it is unknown whether the time of viral clearance is also crucial for the generation of anti-SARS-CoV-2 antibodies. The longer the time of active infection, the higher the levels of plasma IgG anti-S1 and IgG anti-RBD immunoglobulins (p = 0.00070 and p = 0.00064, respectively; Figure 2d).

Correlation between frequencies of TNF-α-producing CD4+ T-cells when non-stimulated (NT) or stimulated with Pep-S (a), Pep-M (b) or Pep-N (c), and the time between PCR+ and PCRneg. PCR+ corresponds to the detection of the SARS-CoV-2 infection, while PCRneg corresponds to the first PCR negative after the infection. Therefore, both dates allow the time for viral clearance to be determined. N=27. (d) Correlation between plasma levels of IgG anti-S1 and anti-RBD (anti-SARS-CoV-2 immunoglobulins) and the time between PCR+ and PCRneg. N=27. Coloured dotted lines estimated threshold of positivity for anti-SARS-CoV-2 immunoglobulin detection. Correlations were assessed using Spearman’s rank correlation with Hockberg’s correction for multiple testing; *adjusted p < 0.05 was considered significant. Each symbol corresponds to an individual.

**Figure 3.**
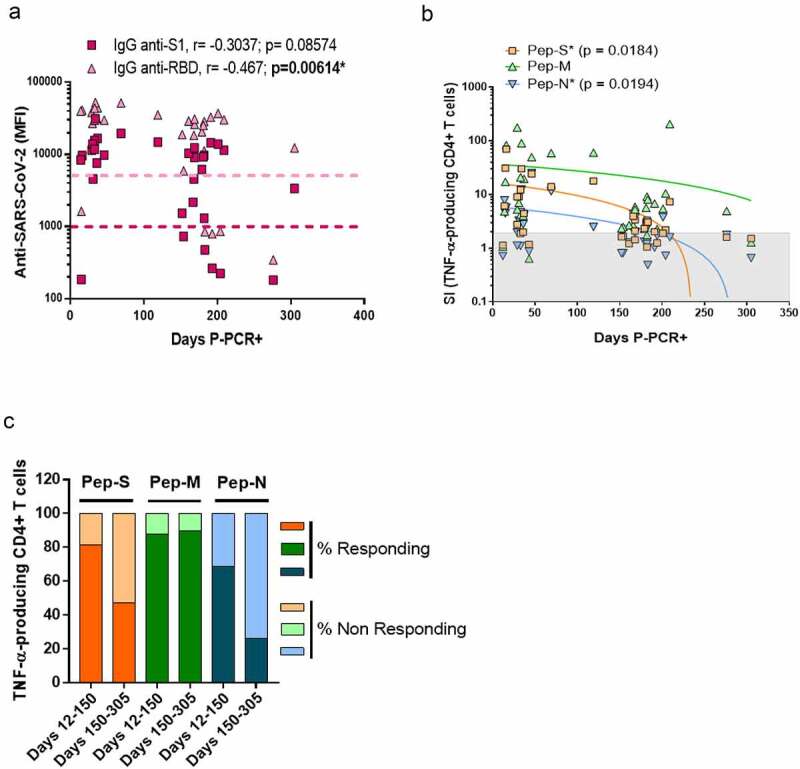
Specific CD4+ T-cell response to SARS-CoV-2 peptides at 10 months post-infection.

We then followed the evolution of anti-SARS-CoV-2 antibodies and specific T-cell responses over time post-infection. Even if IgG anti-S1 and anti-RBD were still detectable in the plasma after 10 months post-infection, their levels were inferior in individuals with a longer recovery period. Specifically, the levels of IgG anti-RBD detection were negatively correlated with days P-PCR+ (p = 0.00614; Figure 3a).

(a) Correlation between the plasma levels of anti-S1 and anti-RBD (anti-SARS-CoV-2 immunoglobulins) and the time between the detection of COVID-19 infection (PCR+) and the time of sample processing (days post-PCR+; P-PCR+). Coloured dotted lines represent the threshold of detection. N=33. (b) Correlation between the SI of TNF-α-producing CD4+ T-cells after stimulation and days P-PCR+. Symbols in the gray zones represent samples with SIs less than 2, indicating non-responding individuals. Correlations were assessed using Spearman’s rank correlation; * p < 0.05 was considered significant. Each symbol corresponds to an individual, N=35. (c) Stacked bars represent the frequencies of individuals with TNF-α-specific T-cell responses corresponding to SIs ≥ 2 (responding) or < 2 (non-responding) when cells were stimulated by Pep-S, Pep-M or Pep-N, in individuals tested 12–150 days P-PCR+ or 150–305 days P-PCR+. N=35.

On the other hand, the SI values of the TNF-α-producing CD4+ T-cells for Pep-S and Pep-N were negatively correlated over time (p = 0.0184 and p = 0.0194, respectively; Figure 3b). We then divided the individuals into two groups: one regrouping individuals 12–150 days P-PCR+ (recent infection, up to 5 months post-infection) and one regrouping individuals 150–305 days P-PCR+ (late infection). In the recent infection group, 81.2%, 87.5% and 68.7% of the individuals responded to Pep-S, Pep-M and Pep-N, respectively, in terms of the frequency of TNF-α-producing CD4+ T-cells. However, the individuals in the late infection group presented 47.4%, 89.5% and 26.3% rates of response to the same peptide pools. Therefore, the frequency of individuals with TNF-α-CD4+ specific memory T-cells against S- or N-derived peptides diminished as time passed after infection (Figure 3c). However, it was encouraging to observe that the frequency of individuals with TNF-α-CD4+ memory against the M-protein remained identical, regardless of the time P-PCR+.

### Individual factors associated with viral clearance and severity of symptoms

**Figure 4.**
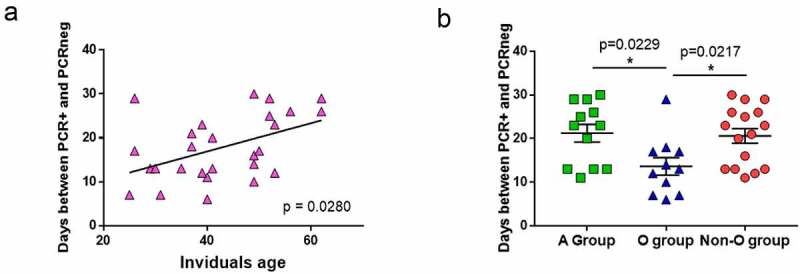
Age and blood groups as factors for viral clearance.

In our study, most of the individuals did not have comorbidities, and almost all had mild symptoms (Table 1). We analyzed whether some other factors associated with infection susceptibility, such as age and ABO group, were related to a better response to SARS-CoV-2. A positive correlation was observed between age and the time needed to reach viral clearance (Figure 4a; p = 0.0280). No correlation was observed between individuals’ age and CD4+ T-cell response (Figure S3a and S3b), anti-spike antibodies levels (Figure S3c and S3d) or absolute lymphocytes’ numbers (CD3+, CD4+ or CD8+ T cells, Figure S3e and S3f).

(a)Correlation between the ages of the individuals and numbers of days between P-PCR+ and PCRneg. Each symbol corresponds to an individual, N=27. (b) Numbers of days between PCR+ and PCRneg in A-group (N=12), inO-group (N=11) and in non-O-group (N=16) individuals. Mann–Whitney U test. *p < 0.05 was considered significant. Correlations were assessed using Spearman’s rank correlation and comparison using Mann-Whitney U tests, both with Hockberg’s correction for multiple testing,

In our study, 40% of the individuals belonged to group O (n = 14), 45.5% to group A (n = 16), 8.5% to group B (n = 3), and 6% to group AB (n = 2). These frequencies were in agreement with those found in the general Spanish population. Due to the low number of individuals having the B and AB blood groups, we regrouped individuals from A, B and AB blood groups (non-O-group). The A-group and non-O-group individuals needed a median of 23 and 22 days, respectively, to reach viral clearance, while the O-group individuals needed a median of 13 days (p = 0.0229 when comparing A-group median from O-group median; Figure 4b).

Then, we analyzed whether the level of anti-A or anti-B immunoglobulins found in the O-group individuals could be related to the severity of COVID-19 symptoms. Using a bead-based assay we assessed ABH subtype specificity and isotype of serum ABO anti-bodies. Of the four asymptomatic patients recruited, three belonged to the O-group, and, even though the number of individuals was low, we observed that the asymptomatic individuals showed higher levels of IgG anti-A (type III) in the plasma than those with mild symptoms (Figure S4a). Additionally, the levels of IgG anti-B (type III and IV) were higher in the O-group individuals with low viral loads, on the day of sample processing (as detected by real-time PCR, high CT), than those with higher viral loads (low CT; Figure S4b). No differences were observed with anti-A I, II, IV, V and VI and anti-B I, II, V, and VI between the studied groups. Nevertheless, this result must be taking with care since the presented N cannot lead to solid conclusions due to the low statistical power. Indeed, significances were lost when multiple testing correction was assessed.

**Figure 5.**
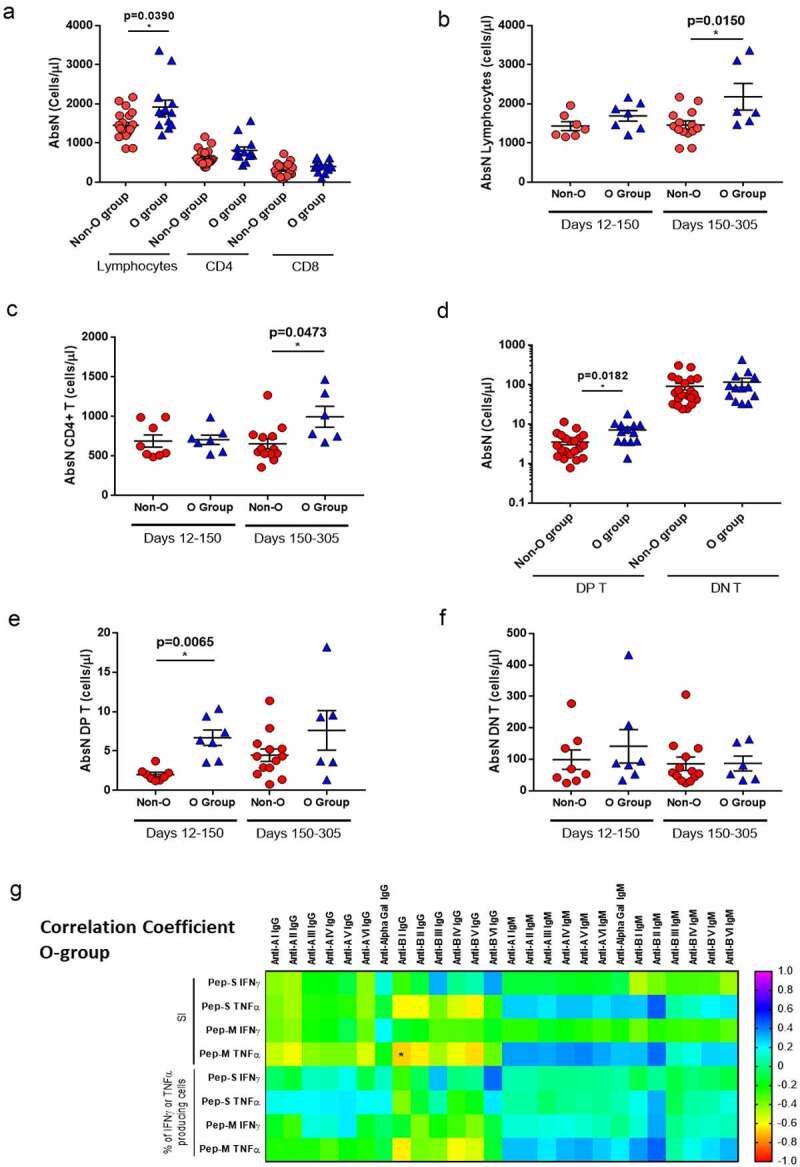
Blood groups as a factor for immune response.

It has previously been observed that the severity of symptoms and viral load are associated with immune dysregulation. We observed that O-group individuals showed higher absolute numbers (AbsN) of total lymphocytes (1862 ± 174 cells/µL, mean ± SEM) than those in the non-O-group (1450 ± 76 cells/µL, mean ± SEM; p = 0.0390; Figure 5a). This can be explained by the lymphopenia already observed in COVID-19 individuals, even those with mild symptoms. Additionally, the individuals from the O-group seemed to recover the AbsN of total lymphocytes after more than 150 days P-PCR+ (1783 ± 143 cells/µL in recent infection group versus 2132 ± 290 cells/µL in late infection group, mean ± SEM; p = 0.0150). Individuals from the non-O-group presented same levels of AbsN lymphocytes after more than 150 days P-PCR+ (1433 ± 113 cells/µL in recent infection group versus 1459 ± 102 cells/µL in late infection group, mean ± SEM; Figure 5b). The same observation was made for the absolute number of CD4+ T cells (Figure 5c).

(a) Whole blood was stained for the determination of the absolute numbers of lymphocytes (AbsN), CD4+ T-cells, and CD8+ T-cells in non-O- (N=21) and O-group (N=13) individuals. (b) Absolute numbers of lymphocytes in non-O- and O-group individuals tested 12–150 days P-PCR+ (N=7 for non-O-group and N=7 for O-group) or 150–305 days P-PCR+ (N=14 for non-O-group and N=6 for O-group). (c) Absolute numbers of CD4+ T cells in non-O- and O-group individuals tested 12–150 days P-PCR+ (N=7 for non-O-group and N=7 for O-group) or 150–305 days P-PCR+ (N=14 for non-O-group and N=6 for O-group). (d) Absolute numbers of CD4/CD8 double-positive (DP T) or double-negative T cells (DN T) in non-O- and O-group individuals. Absolute numbers of CD4/CD8 double-positive (DP T, e) or double-negative T cells (DN T, f) in non-O- and O-group individuals tested 12–150 days P-PCR+ (N=7 for non-O-group and N=7 for O-group) or 150–305 days P-PCR+ (N=14 for non-O-group and N=6 for O-group). (g) Heat map of Spearman correlation coefficients for indicated features in O-group individuals (N=14). Spearman’s correlation and Mann–Whitney U tests were employed using Hochberg’s correction for multiple testing. *p < 0.05 was considered significant.

Other lymphocytes subset was found significantly different between the O- and the non-O-group, the CD4+ CD8+ double-positive T cell subset (DP T, Figure 5d). There was no significant difference in the absolute counts of DP T cells in O-group individuals between recent and late infection groups (Figure 5e). However, the absolute count of DP T cells in the recent infected non-O group (2.01 ± 0.28 cells/µL, mean ± SEM) was significantly lower than the O-group (6.70 ± 0.98 cells/µL, mean ± SEM; p = 0.0065). No difference was observed for the CD4/CD8 double-negative (DN T) T cell subset (Figure 5f).

Therefore, ABO grouping might influence the course of infection. Indeed, negative correlations were observed between the levels of anti-B immunoglobulins and the level of TNF-α-producing CD4+ T-cell responses, when activated with Pep-M, in the O group (Figure 5g), indicating that, the lower the anti-B type I immunoglobulins in O-group individuals, the higher the generation of specific immune responses. Nevertheless, due to the low numbers of evaluated individuals, additional studies must be done to increase the statistical power and make more consistent conclusions.

### ABO group and anti-SARS-CoV-2-specific immune response

**Figure 6.**
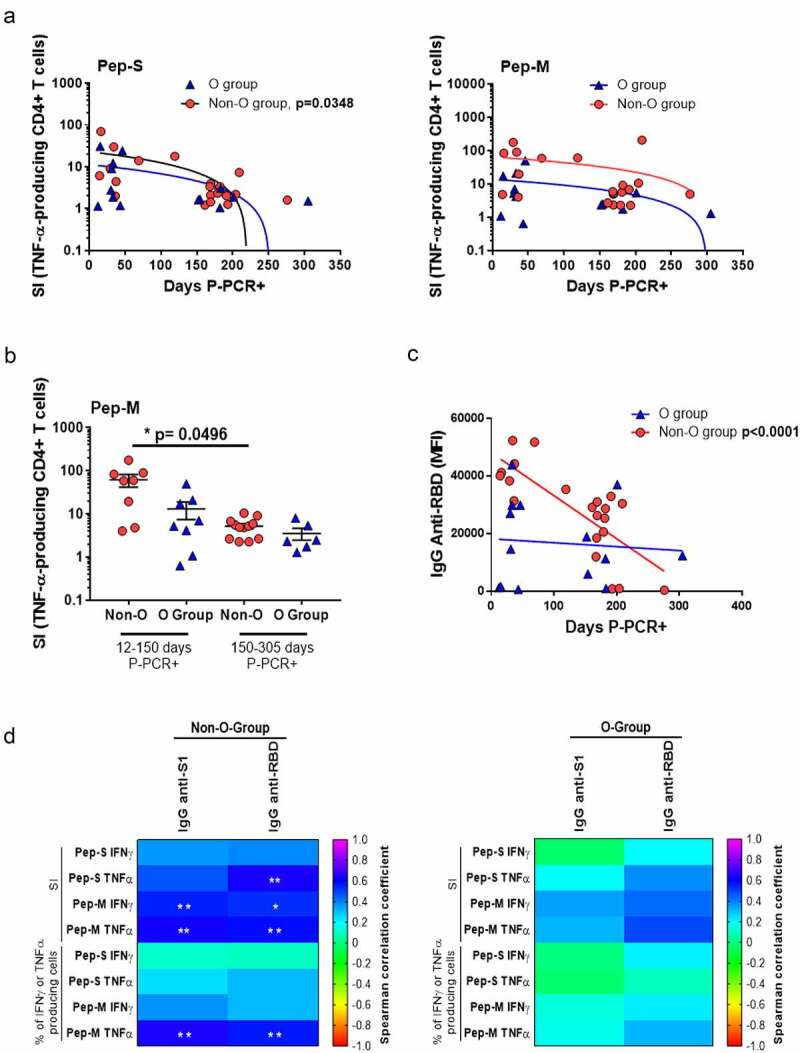
Blood groups as a factor for specific CD4+ T-cell response.

As a low AbsN of lymphocytes can cause dysregulation in the immune response, we studied whether the blood group could influence the generation of anti-SARS-CoV-2 memory in the long term post-infection. When cells were stimulated with Pep-M, the frequencies of IFN-γ- and TNF-α-producing CD4+ T-cells were significantly higher in group A than in group O (p = 0.0085 and p = 0.0245, respectively; Figure S5). In addition, when cells were stimulated with Pep-S, the SI of TNF-α-producing CD4+ T-cells in individuals with recent infection was higher than individuals with late infection regarding the negative correlation between SI and days P-PCR+ (p = 0.0348) in the non-O-group individuals, but not in the O-group (Figure 6a). No correlation between SI and days P-PCR+ was observed for the Pep-M, nevertheless, the frequencies of individuals with TNF-α-producing CD4+ T-cell responses were lower in the later infection non-O-group than in the recent infection non-O-group (p = 0.0496; Figure 6b). Similar results were observed when correlating the days P-PCR+ and level of anti-RBD in the plasma in the non-O-group individuals (p < 0.0001; Figure 6c). The frequencies of individuals with high specific responses and with high humoral anti-SARS-CoV-2 levels were inferior as time passed from infection in the non-O-group but not in the O-group, showing that a strong but labile anti-SARS-CoV-2 immune response could occur in the non-O-group.

(a) Correlations between SI of TNF-α-producing CD4+ T-cells when stimulated with Pep-S (left panel) and Pep-M (right panel) in O-/non-O-groups (N=14/21) and days P-PCR+. (b) Frequencies of TNF-α-producing CD4+ T-cells, when stimulated with Pep-M in O-/non-O-group individuals, tested 12–150 days P-PCR+ (N=8/N=8) or 150–305 (N=6/N=12) days P-PCR+. (c) Correlations between level of anti-RBD immunoglobulins and days P-PCR+. Each symbol corresponds to an individual (N=21 for non-O-group and N=14 for O-group). (d) Heat map of Spearman correlation coefficients of indicated features in non-O-group (left panel, N=21) and O-group (right panel, N=14) individuals. Correlations were assessed using Spearman’s rank correlation with Hochberg’s correction for multiple testing. *p < 0.05; **p < 0.01.

Finally, we checked whether there were correlations between the cellular and humoral specific responses. Positive correlations between the levels of anti-SARS-CoV-2 immunoglobulins and specific cellular responses were observed in non-O-group individuals but not in O-group individuals, likely indicating a coordinated cellular and humoral immune response in the non-O-group individuals (Figure 6d).

In summary, the ABO blood group might be an essential factor influencing the time of viral clearance and the intensity of the TNF-α-associated response over time post-infection and further studies with additional evaluated individuals could confirm such results. The non-O- group showed the highest TNF-α-associated response, as well as a significantly less frequent response after 10 months post-infection.

## Discussion

In this study, we aimed to evaluate the induction and duration of T-cell specific memory and humoral immunity in individuals who had recovered from SARS-CoV-2 infections with asymptomatic/mild symptoms individuals who represent the great majority of infected subjects. We also studied some susceptibility factors that may be associated with the development of sustained immunity. At 10 months post-infection, almost all the enrolled individuals were still positive for SARS-CoV-2-specific IFN-γ- and TNF-α-producing CD4+ T-cells against the M viral protein and for anti-S1 or anti-RBD immunoglobulins. However, we also observed a decreased frequency of individuals with SARS-CoV-2-specific responses against the S or N viral proteins with the time passed since the infection, as had already been observed in other studies [28,29]. In addition to a decreased immunity developed against the SARS-CoV-2 S-protein, we observed that the CD8+ T-cell memory response seemed to be deficient. Whether a robust CD8+ T response might be generated is a worthwhile question, as the CD8+ cytotoxic T-cell response is generally profoundly implicated in viral clearance. It has been shown that COVID-19 subjects present elevated Th2-cytokines (IL-4 or IL-10), which could inhibit the Th1 response [30]. It has already been described that Th1 participates in the activation, proliferation, and differentiation of the cytotoxic memory CD8+ T-cells [31]. Low CD8+ T-cell activation due to an inadequate Th1 response and high Th2 response could explain the low CD8+ T-cell response frequency. Therefore, it would be interesting to design vaccines against non-spike proteins along with the S-protein, which could promote the generation of stronger CD4+ and CD8 + T-cell memory.

Looking for factors that could influence the frequency of response against SARS-CoV-2-derived peptides, we observed that the age of the subjects was important for rapid viral clearance. Older individuals are more susceptible to developing severe COVID-19 symptoms, due to several factors such as a decline in organ function, basal inflammation, or the presence of comorbidities [32,33]. Nevertheless, we report that age is also associated with viral clearance for the first time in this paper, to the best of our knowledge.

ABO blood groups have been implicated in the susceptibility to and severity of SARS-CoV-2 infections [34,35]. In particular, the O-group has been associated with a diminished risk of acquiring COVID-19 and developing severe disease, compared to non-O-group [36–38]. In this study, we showed that blood groups might influence the generation of memory T-cell responses for the first time. We showed that non-O-group subjects needed more time to clear the virus than the O group, and that they presented higher frequencies of IFN-γ and TNF-α CD4+ T-cell responses against Pep-M than O-group individuals. Moreover, there were positive correlations between the humoral and cellular specific responses only in non-O-group individuals but not in O-group individuals. These results might indicate that the non-O-group can develop a more robust response against Pep-M; we hypothesize that this could be due to the non-O-group individuals needing more time to clear the virus. Thus, the longer the time of viral exposure, the stronger the response, even in mild COVID-19 cases. In severe COVID-19 cases, patients achieve a higher adaptive immune response, where a more elevated and/or extended viral load could be related to the higher immune response [39]. On the other hand, the frequency of non-O-group individuals showing TNF-α-related T-cell memory responses and plasma levels of anti-Spike immunoglobulins were significantly lower after a long time P-PCR+ than the O-group subjects. Thus, even though the memory T-cell response was initially higher in the non-O-group, the frequency of individuals with a TNF-α response seemed to be lost over time, as indicated as well by the plasma humoral anti-SARS-CoV-2 immunoglobulins, showing a deterioration of the sustainability of the specific immune response. These results should be confirmed by further studies with additional analyzed individuals in order to increase the statistical power.

One characteristic of COVID-19 is lymphopenia, even in individuals with mild symptoms, with more profound lymphopenia in patients with severe symptoms [32]. We observed that the non-O-group presented significantly lower absolute numbers of total lymphocytes than the O-group. On the contrary, the O-group showed higher absolute counts of highly DP T cells than the non-O-group. These DP T cells were already implicated in enhancing cytotoxic responses during viral infections [40]. These results might indicate that the immune systems in the non-O-group subjects were more affected by the infection than the O group’s immune systems. According to the literature, the O-group seems to be associated with a lower risk of acquiring COVID-19 than non-O groups [35]. The principal factor relating the ABO group to COVID-19 susceptibility may be the presence of anti-A, anti-B or anti-glycan antibodies such as anti-Gal or anti-N-Glycolyl neuraminic acid [41,42]. The presence of such antibodies especially in O-group individuals could inhibit the SARS-CoV-2 S protein’s adhesion to ACE2-expressing cell lines, as has already been observed for SARS-CoV [43]. Indeed, due to the presence of SARS-CoV-2 entry receptors on the epithelial cells of the upper respiratory tract, these cells can be infected and produce newly synthesized viral particles. Because spike protein has various glycosylation sites, this protein can be glycosylated by the glycan’s related to the infected cells and therefore can carry at their surface ABO-related epitopes [41], as already seen in SARS-CoV-1 [43]. Moreover, epithelial cells (from the kidney) have been shown to express A subtypes II, III and IV [22]. If similarly expressed in the airway, they could be potentially acquired by SARS-CoV-2 viruses upon infection of epithelial cells. This hypothesis is also based on the fact that ABH antigen subtypes have been described to be expressed differentially into secretor and non-secretor epithelial cells in human lung airways [44,45]. Therefore, SARS-CoV-2 harboring ABO antigens could be possibly neutralized by the anti-A or anti-B antibodies, both being present in O-group individuals, which could bring some protective role against infection and replication. These studies resonate with our results showing that asymptomatic individuals or with low viral load had higher levels of anti-A and anti-B in plasma in O-group individuals. Nevertheless, we must emphasized that these results were based on N=3 (individuals with asymptomatic disease or low viral load) versus N11 (individuals with mild symptoms or low CT in PCR). Therefore, even though previous studies are in line with our preliminary results, more individuals should be analyzed before concluding about ABO-associated antibodies and the severity of COVID-19 symptoms. Thus, further study will be required regarding the role of ABO antibody subtype specificity in COVID-19 infection.

Therefore, one can hypothesize that such virus blocking may lower the infectious viral load in O-group subjects; then, that the lower viral load associated with the potential protective effect of anti-blood-group antibodies in an O-group subject accelerates the clearance of the virus and reduces its impact on the immune system. However, the implications of other factors, such as-yet-unknown factors related or unrelated to ABO blood groups that could potentially affect the susceptibility to SARS-CoV-2 infection, cannot be discarded.

Taken together, these results confirmed the existence of SARS-CoV-2-specific CD4+ T-cell and humoral responses in the majority of the individuals who had recovered from COVID-19 at 10 months post-infection. However, the response generated by the virus was identified as a predominantly CD4+ T-cell over CD8+ T-cell response, with more robust responses against M- or S-peptide pools over Pep-N. A more in-depth analysis demonstrated that the intensity of the humoral and memory T-cell response might be related to the ABO blood group and age. Therefore, determining the individual characteristics that may influence the immune response to SARS-CoV-2 must be considered for the future design of vaccines with long-term efficacy.

## Supplementary Material

Supplemental Material

## Data Availability

The authors confirm that the data supporting the findings of this study are available within the article and its supplementary materials.
