# Supplementary material for: ABO blood group is involved in the quality of the specific immune response anti-SARS-CoV-2
Source: Virulence. 2021 Dec 30;13(1):30–45. doi: 10.1080/21505594.2021.2019959 (PMC9794011; doi:10.1080/21505594.2021.2019959)
Supplement: Supplemental Material [file KVIR_A_2019959_SM6451.zip › supplementary/Supplemental_Data.docx]

**ABO blood group is involved in the quality of the specific immune response anti-SARS-CoV-2**

Sergio Gil-Manso1, Iria Miguens Blanco2, Bruce Motyka3, Anne Halpin3,4, Rocio Lopez-Esteban1, Veronica A. Perez-Fernandez1, Diego Carbonell1,5, Luis Andrés López-Fernández6, Lori West3,7, Rafael Correa-Rocha1*, and Marjorie Pion1*

1Laboratory of Immune-Regulation, Gregorio Marañón Health Research Institute (IiSGM), Gregorio Marañón University General Hospital, Madrid, Spain

2Department of Emergency, Gregorio Marañón University General Hospital, Madrid, Spain

3Depts. of Pediatrics, Alberta Transplant Institute and Canadian Donation and Transplantation Research Program; University of Alberta, Edmonton, Alberta, Canada

4Laboratory Medicine & Pathology, University of Alberta, Edmonton, Alberta, Canada

5Department of Hematology, Gregorio Marañón University General Hospital, Madrid, Spain

6 Service of Pharmacy, Gregorio Marañón Health Research Institute (IiSGM), Gregorio Marañón University General Hospital, Spanish Clinical Research Network (SCReN), Madrid, Spain

7Medical Microbiology & Immunology, Surgery, and Laboratory Medicine & Pathology; University of Alberta, Edmonton, Alberta, Canada

*To whom correspondence should be addressed: rafael.correa@iisgm.com; marjorie.pion@iisgm.com. Phone: 0034 913442854

Address: Laboratorio de InmunoRegulacion- Edificio de Medicina y Cirugía Experimental, planta baja- Hospital General Gregorio Marañon- C/ Máiquez 9, 28009, Spain

**Supplemental DATA**


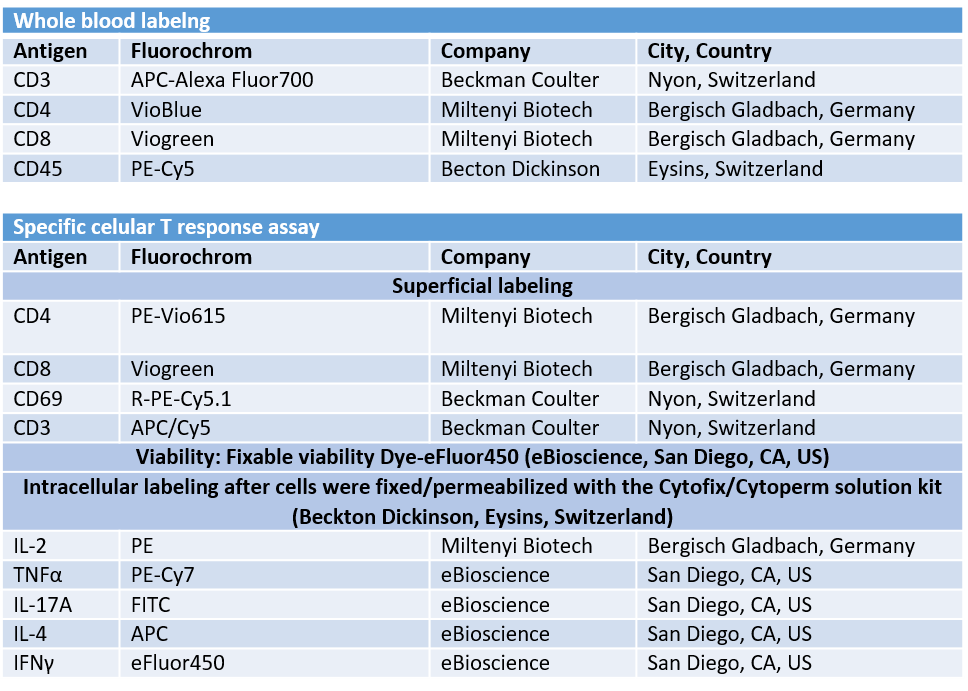


Supplemental Table 1

**Supplemental Table 1: antibodies used in flow cytometry analysis**


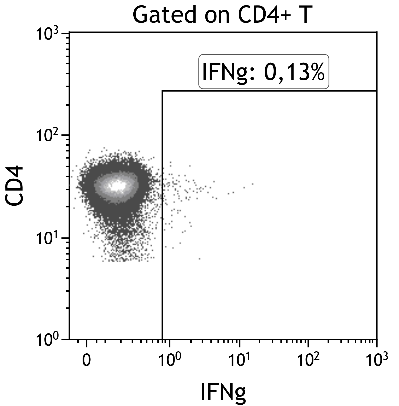

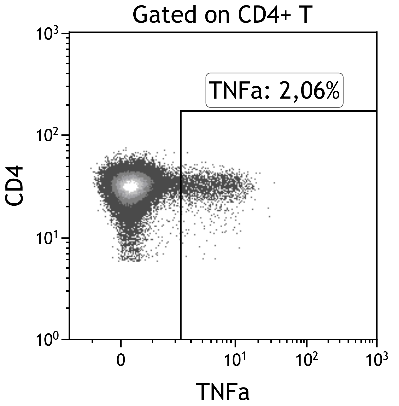

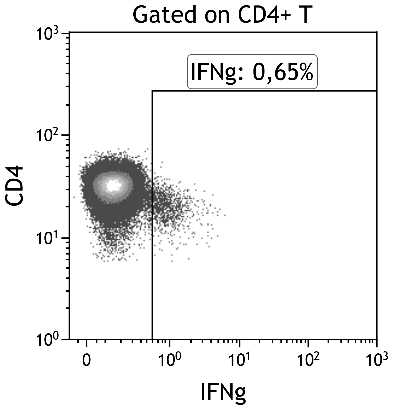

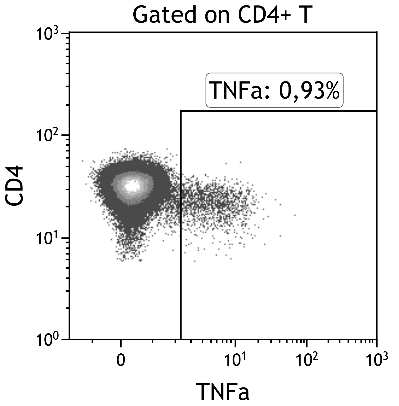

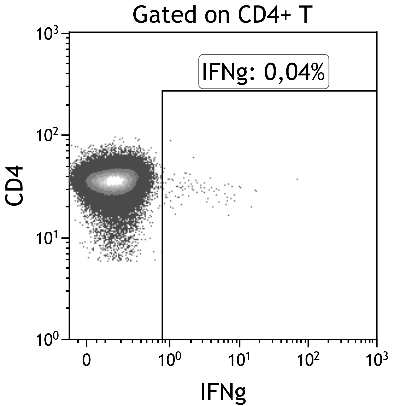

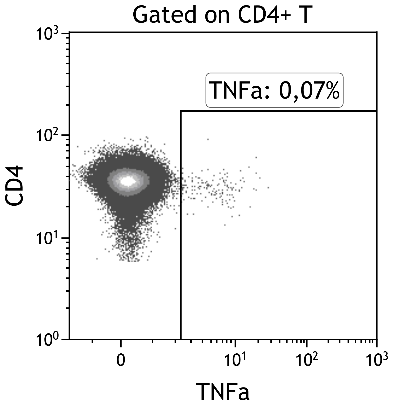

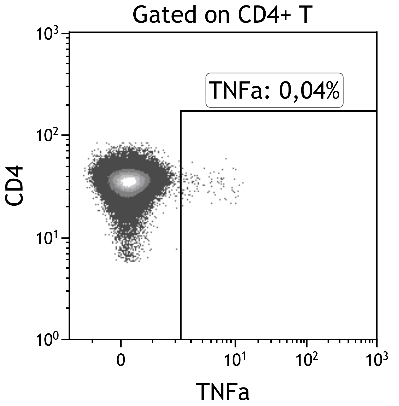

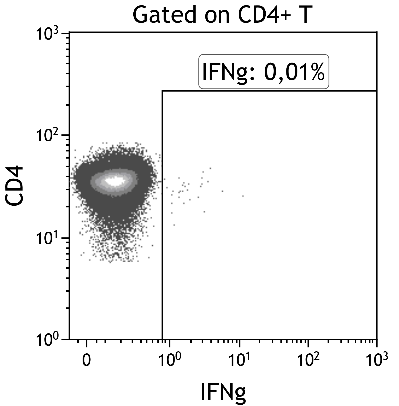

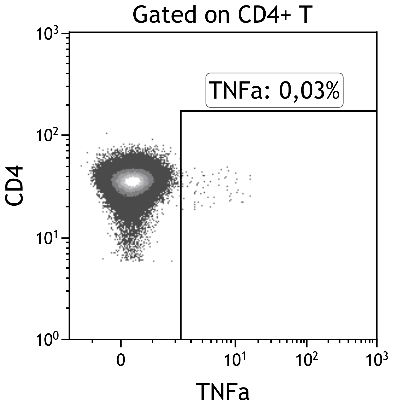

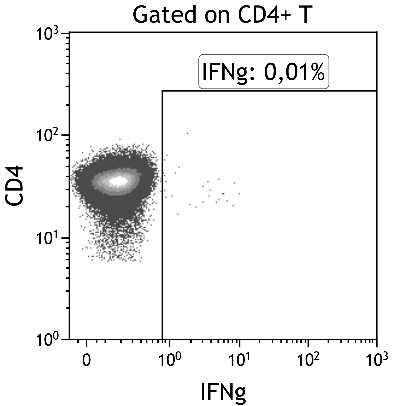

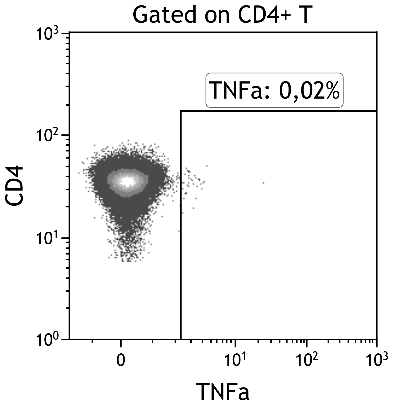

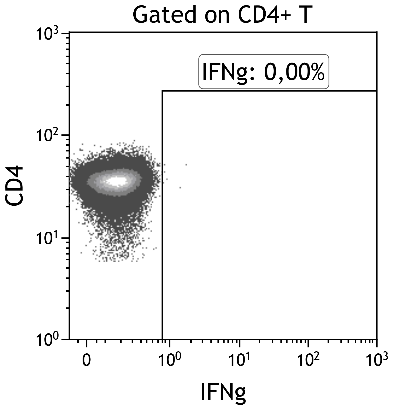


**Supplemental Figure 1: Specific CD4+ T-cell responses to SARS-CoV-2**

Representative examples of flow cytometry plots of specific IFN-γ- and TNF-α-producing CD4+ T cells for non-treated condition (NT) or after 6h of stimulation with Pep-S, Pep-M, Pep-N, Pep-CMV and CytoStim.

CytoStim

Pep-CMV

Pep-M

Pep-N

Pep-S

Supplemental Figure 1

NT

C

B

A


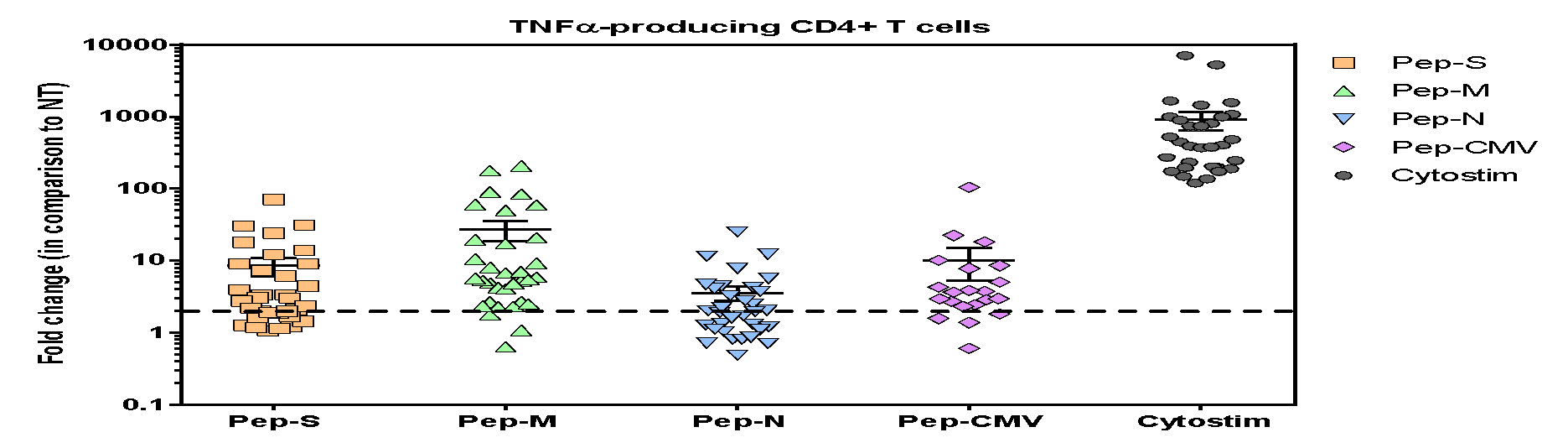

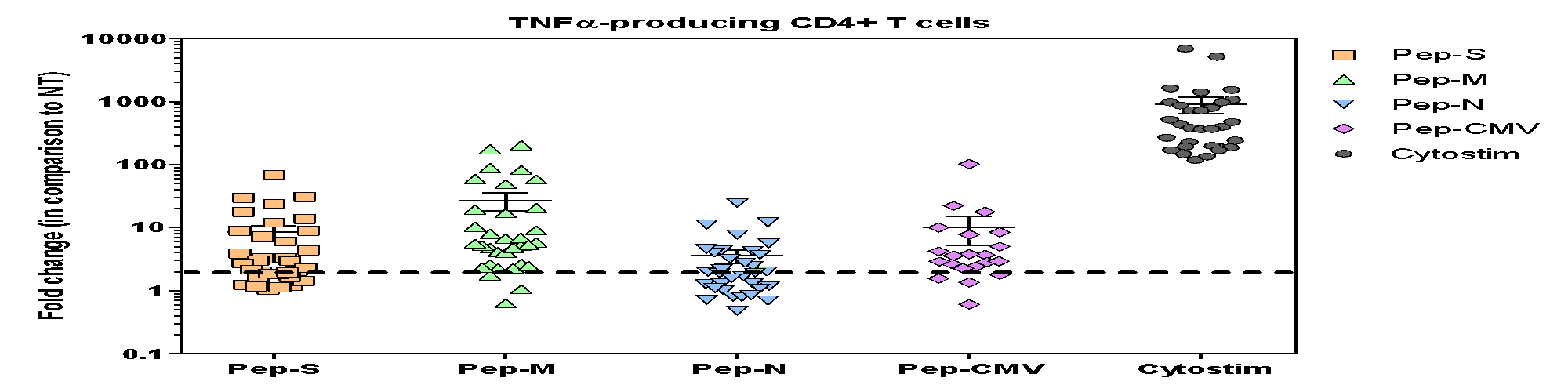

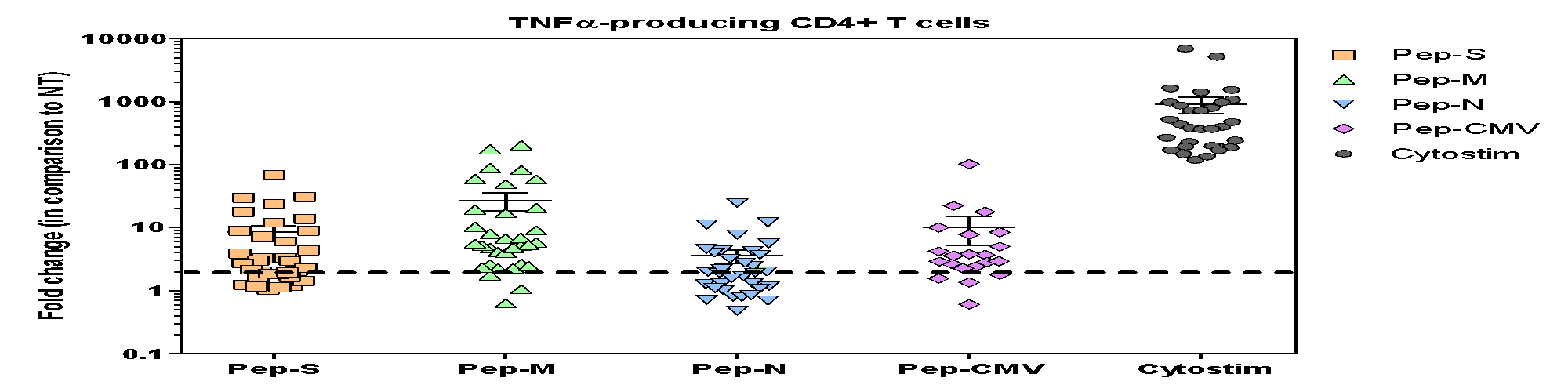

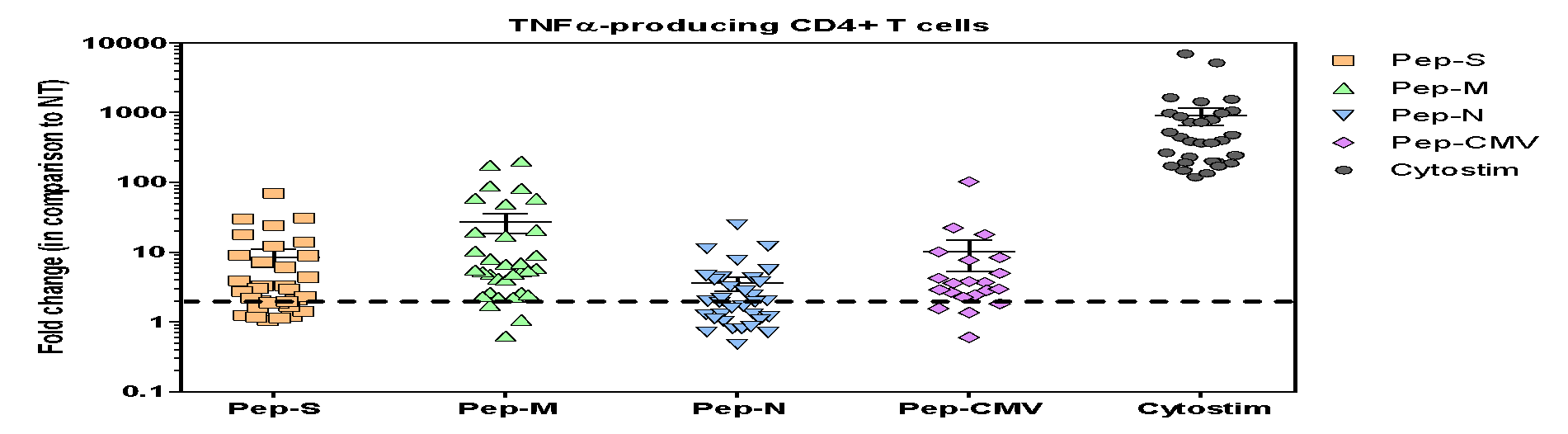

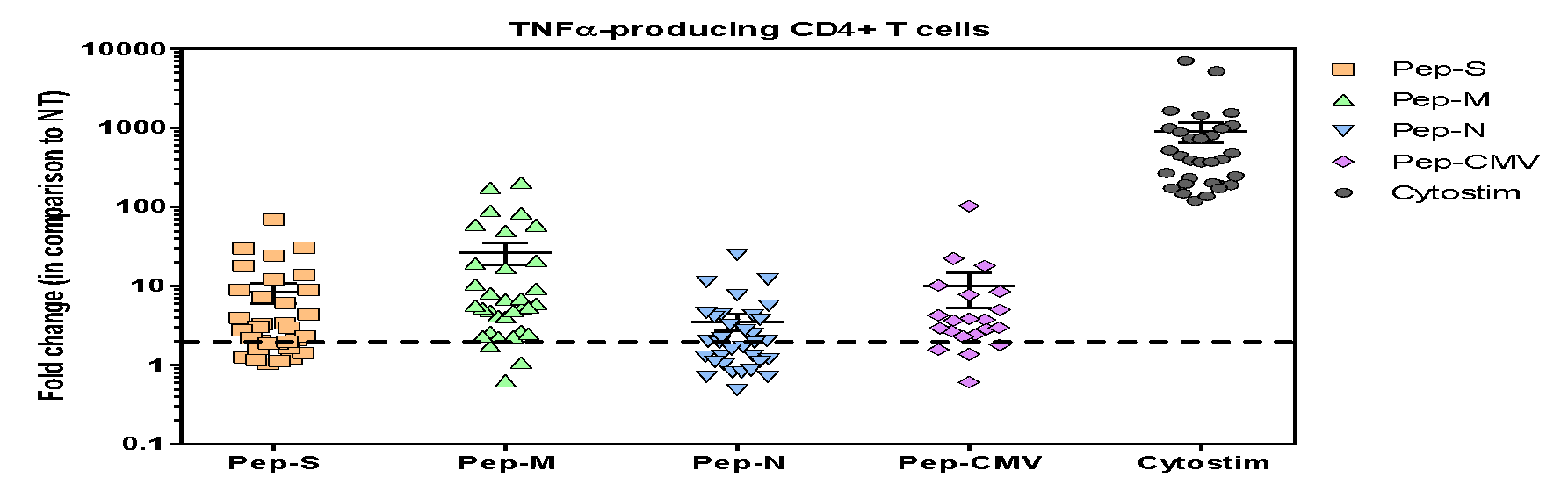

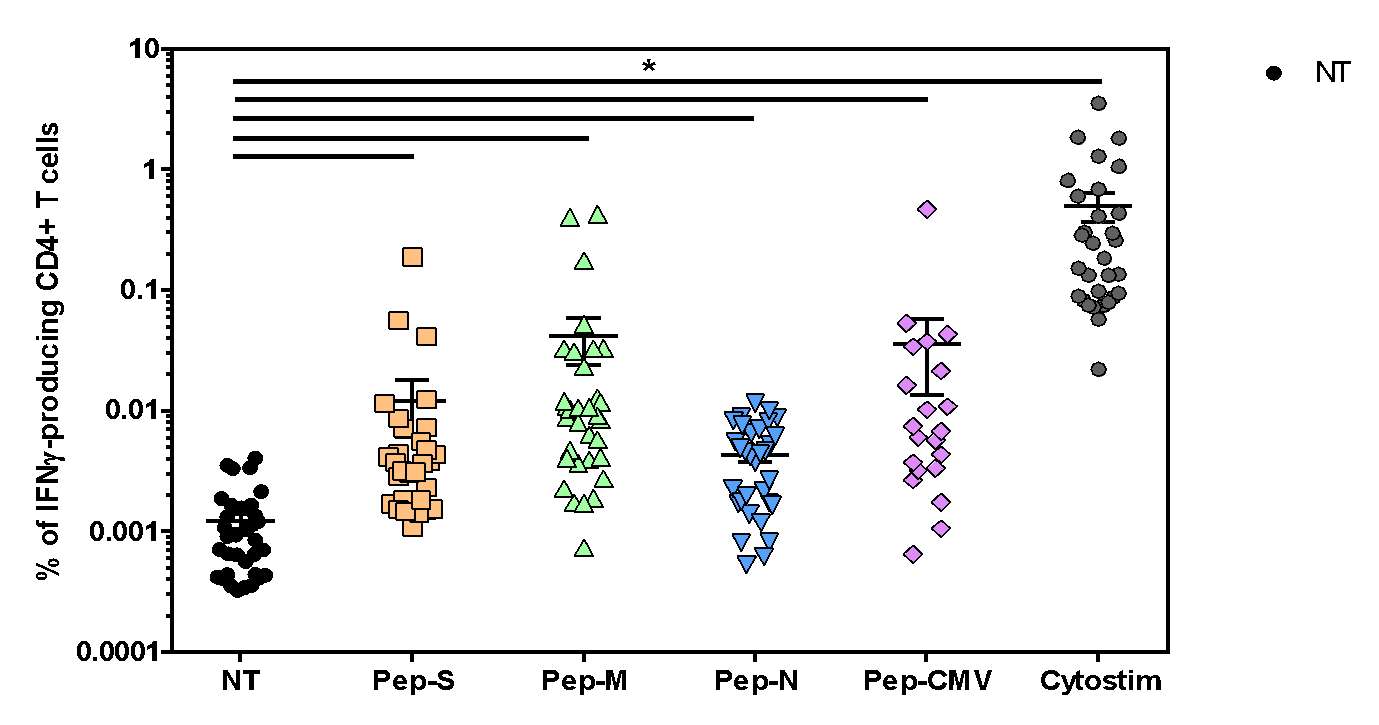


Supplemental Figure 2

**Supplemental Figure 2: TNF-α, IFN-γ, IL-17A, IL-2, and IL-4-specific T-cell responses to SARS-CoV-2-derived peptides pools**

PBMCs were isolated and stimulated by SARS-CoV-2-derived peptide pools (Pep-S; Pep-M and Pep-N), with CMV-derived peptides (Pep-CMV) or with CytoStim. After 6h of activation, cells were surface labeled and stained intracellularly. (A) Stimulation Index (SI) for intracellular IFN-γ and TNF-α-producing CD4+ T cells. (B) Stimulation Index (SI) for intracellular IL17-A, IL-2 and IL-4-producing CD4+ T cells (left panels) or CD8+ T cells (right panels). (C) Stimulation Index (SI) for intracellular IFN-γ and TNF-α-producing CD8+ T cells. SI for each subjects was calculated dividing the frequency of cytokine-producing CD4+ / CD8+ T cells in stimulated condition (Peptides or CytoStim) and frequency of cytokine-producing CD4+ / CD8+ T cells in the non-treated condition (NT). Each symbol corresponds to an individual. The grey area represents the stimulation index inferior at 2 that is considered as a negative response to the stimulation in comparison to NT.

Supplemental Figure 3

**A**

**C**

**E**

**B**

**D**

**F**

**Supplemental Figure 3: Individuals’ age is not a factor for anti-SARS-CoV-2 responses**

(A) Correlations between individuals’ age and stimulation index (SI) calculated for the TNF-α-producing CD4+ T cells when cells were stimulated with Pep-S, Pep-M or Pep-N. (B) SI for the TNF-α-producing CD4+ T cells when cells were stimulated with Pep-S, Pep-M or Pep-N after grouping individuals based on age; ≤ or > 40 years old (yo). (C) Correlations between individuals’ age and anti-SARS-CoV-2 antibodies (Mean Fluorescence Intensity; MFI). Coloured dotted lines represent the threshold of detection. (D) Anti-SARS-CoV-2 antibodies (MFI) in individuals grouped based on age; ≤ or > 40 years old (yo). (E) Correlations between absolute numbers (AbsN) of Lymphocytes, CD3, CD4+ or CD8+ T cells and individuals’ age. (F) Absolute numbers (AbsN) of Lymphocytes, CD3, CD4+ or CD8+ T cells in individuals grouped by age; ≤ or > 40 years old (yo). Each symbol corresponds to an individual. correlations were perfomed using the Spearman Correlation Test, corrected by Hockberg’s procedure. *p<0.05 was considered as significant.


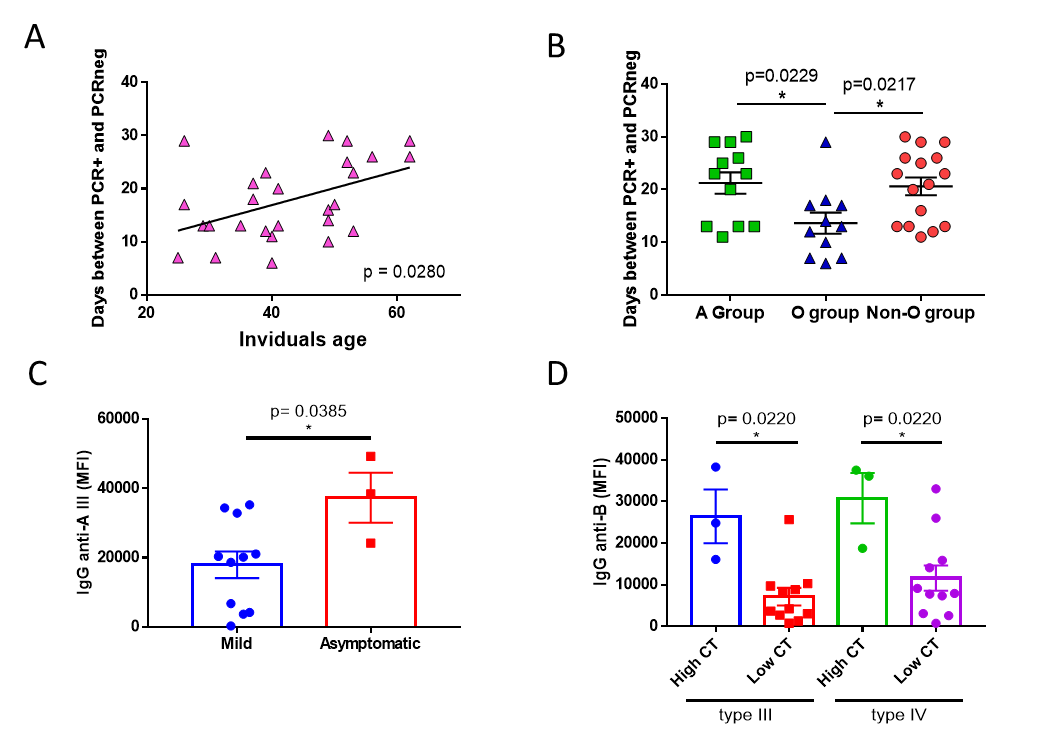


**Supplemental Figure 4: Anti-A and Anti-B immunoglobulins in O-group individuals**

(A) Plasma levels in O-group individuals of anti-A type III immunoglobulins in mild (N=11) and asymptomatic (N=3) COVID-19 individuals. (B) Plasma levels in O-group individuals of anti-B type III and IV immunoglobulins in individuals presenting high (N=3) and low CT (N=11) in the real-time PCR the day of sample processing in mild and asymptomatic COVID-19 individuals. *p < 0.05 was considered significant. Correlations were assessed using Spearman’s rank correlation and comparison using Mann-Whitney U tests, both with Hockberg’s correction for multiple testing,

Supplemental Figure 4

**A**

**B**

Supplemental Figure 5

**Supplemental Figure 5: Blood groups as factor for CD4+ T cell specific TNF-α and IFN-γ responses**

Frequencies of IFN-γ- and TNF-α-producing CD4+ T cells in non-treated (NT) or stimulated PBMC with Pep-S; Pep-M; Pep-N and Pep-CMV in ABO groups. Symbols on the dotted line represent the frequency of specific CD4+ T cell with negative results. Mann-Whitney U tests. *p<0.05 was considered as significant.
